# Probiotics Isolated From Animals in Northwest China Improve the Intestinal Performance of Mice

**DOI:** 10.3389/fvets.2021.750895

**Published:** 2021-09-27

**Authors:** Yingying Li, Dan Jia, Jiahui Wang, Hehai Li, Xijuan Yin, Junlong Liu, Jinming Wang, Guiquan Guan, Jianxun Luo, Hong Yin, Sa Xiao, Youquan Li

**Affiliations:** ^1^State Key Laboratory of Veterinary Etiological Biology, Key Laboratory of Veterinary Parasitology of Gansu Province, Lanzhou Veterinary Research Institute, Chinese Academy of Agricultural Sciences, Lanzhou, China; ^2^College of Veterinary Medicine, Northwest A&F University, Xianyang, China; ^3^Jiangsu Co-innovation Center for Prevention and Control of Important Animal Infectious Diseases and Zoonoses, Yangzhou, China

**Keywords:** *Bacillus velezensis*, *Lactobacillus plantarum*, *Lactobacillus salivarius*, immunity, intestinal flora

## Abstract

Antibiotic resistance is an increasingly prevalent problem worldwide. Probiotics are live microorganisms that provide health benefits to human beings and animals and also antimicrobial activity against pathogens and might be an antibiotic alternative. The gastrointestinal tract of animals can be a suitable source of finding novel antimicrobial agents, where the vast majority of gut microbes inhabit and a plurality of antimicrobial producers exhibit either a wide or narrow spectrum. Animals that live in Northwest China might possess a special commensal community in the gut. Therefore, the purpose of this study was to assess the effects of three probiotic strains (including *Lactobacillus salivarius* ZLP-4b from swine, *Lactobacillus plantarum* FBL-3a from beef cattle, and *Bacillus velezensis* JT3-1 from yak), which were isolated from livestock in this area, on the overall growth performance, immune function, and gut microbiota of mice. The results showed that the *L. salivarius* ZLP-4b group not only improved the growth performance but also amended the intestinal mucosa morphology of mice. Furthermore, the supplementation of *L. plantarum* FBL-3a and *L. salivarius* ZLP-4b strains significantly increased the content of anti-inflammatory cytokines IL-4 and IL-10 but decreased the pro-inflammatory factor IL-17A. The levels of pro-inflammatory factors IL-6, IL-17A, and TNF-α were also decreased by the *B. velezensis* JT3-1 group pretreatment. The 16S rDNA sequence results showed that the probiotic administration could increase the proportion of Firmicutes/Bacteroidetes intestinal microbes in mice. Furthermore, the relative abundance of *Lactobacillus* was boosted in the JT3-1- and ZLP-4b-treated groups, and that of opportunistic pathogens (including Proteobacteria and Spirochaetes) was diminished in all treated groups compared with the control group. In conclusion, *B. velezensis* JT3-1 and *L. salivarius* ZLP-4b supplementation enhanced the overall performance, intestinal epithelial mucosal integrity, and immune-related cytokines and regulated the intestinal microbiota in mice.

## Introduction

Antibiotic has been applied for almost 100 years as a predominant strategy in controlling infectious diseases and improving the growth performance of animals (1). Along with the inappropriate and excessive use of antibiotics, antibiotic resistance issues have been gradually exposed (2). There is an increasing number of bacteria which have developed drug resistance, such as *Salmonella* with multidrug resistance (3). Besides this, antibiotic resistance is also found in association with gut microbiota disturbance. Furthermore, dysbiosis of microbiota might cause numerous diseases, including obesity, autoimmune diseases, allergy, and intestinal diseases such as irritable bowel syndrome (IBD) (4). Consequently, alternatives to antibiotic treatment were required to be explored (5).

Probiotics have been proven to confer benefits to human and animals within reasonable adoption (6). At present, major probiotics can be classified into *Lactobacillus* spp. (such as *Lactobacillus rhamnosus* and *Lactobacillus acidophilus*), *Bacillus* spp. (*Bacillus subtilis*), *Bifidobacterium* spp. (*Bifidobacterium longum* and *Bifidobacterium animal*), yeast (*Saccharomyces cerevisiae*) and *Clostridium* spp. (*Clostridium butyricum*), and so on (7). In addition, next-generation probiotics (*Akkermansia muciniphila, Faecalibacterium prausnitzii*, and *Eubacterium hallii*) and genetically modified probiotics (GM probiotics: mutation and overexpression) were vigorously researched (8, 9). The basic mechanism by which probiotics exert beneficial effects is explained as follows: (1) colonization and restoration of disordered intestinal microbiota in the host, (2) competitive exclusion of harmful microbes and antimicrobial molecule production, (3) cell antagonism, cell adhesion, and mucin expression, and (4) regulation of innate and acquired immunity of the host (10). Studies demonstrated that *B. subtilis* supplementation could ameliorate heat-induced behavioral and inflammatory reactions (11). Sun et al. (12) reported that *Bifidobacterium* administration altered the commensal community in the gut of mice and regulated the mucosal immunity as determined by Tregs in the colitis model under the cytotoxic T lymphocyte-associated protein 4 blockade conditions. Li et al. (13) also found that *Bacillus* spp. isolated from feces of yaks significantly impacts the growth performance, the action of intestinal digestive enzymes, immune responses, and antioxidative capacity in mice. In addition, *Lactobacillus casei* overproducing conjugated linoleic acids illustrated a significant protective effect on *Salmonella enteric* serovar *Typhimurium* challenge (14).

Xinjiang and Gansu are two provinces of Northwest China, which are characterized by a rough terrain and harsh weather. Consequently, livestock inhabiting in the alpine areas are very resilient to harsh ecological and climatic environmental changes. Thus, altitude hypoxia (low temperature and thin air) would put constant heat on the process of evolution (15). A previous study showed that environmental factors helped to shape the components and functions of the intestinal microbiota in people who lived at high altitudes (16). Fan et al. (17) demonstrated that altitude impacted on the diversity of microbes and herbage fermentation in the rumen of yaks. This hinted that the animals that lived in Northwest China might possess a special commensal community in the gut compared to those in the plain region. However, there are few research about the gut microbiota of these animals, and studies on probiotics existing in these animals are also seldom reported.

Therefore, this study set out to assess the effects of *Bacillus* spp. (*Bacillus velezensis* JT3-1 obtained from yak) and the effects of two strains of *Lactobacillus* (*Lactobacillus plantarum* FBL-3a sourced from beef cattle and *Lactobacillus salivarius* ZLP-4b isolated from swine) on the growth performance, intestinal morphology, immune-related cytokines, and gut microbiota of mice.

## Materials and Methods

### Probiotic Strains and Animal Experiments

The *B. velezensis* JT3-1 used in this experiment was obtained from feces of healthy domestic yak (*Bos grunniens*) in Gansu province of China. Two strains of *Lactobacillus* were also isolated from healthy animal feces in Northwest China and included *L. plantarum* FBL-3a isolated from feces of beef cattle in Xinjiang Uygur Autonomous Region and *L. salivarius* ZLP-4b obtained from feces of swine in Gansu province. Our previous results found that these probiotics exhibited good tolerance and antimicrobial activity. The antibiotic sensitivity experiments, resistance gene tests, and hemolytic experiments conducted showed the safety of the JT3-1, FBL-3a, and ZLP-4b strains. Based on this, the complete genome sequence of *B. velezensis* JT3-1 (GenBank: CP032506), *L. plantarum* FBL-3a (GenBank: CP034694), and *L. salivarius* ZLP-4b (GenBank: CP062071) was performed using a PacBio Sequel sequencing platform at Beijing Genewiz Bioinformatics Technology Co., Ltd. *B. velezensis* JT3-1 was cultured in nutrient agar medium at 37°C for 24 h aerobically. *Lactobacillus* was cultured at 37°C for 24 h under anaerobic environment on MRS agar medium. Then, the bacterial cells of these strains were evaluated by using the plate count method.

The 2-week-old female Kunming mice (*n* = 48) were obtained from Lanzhou Veterinary Research Institute, Chinese Academy of Agricultural Sciences and raised in specific pathogen-free conditions. They were randomly and equally assigned to four groups, including the control group and the JT3-1-, FBL-3a-, and ZLP-4b-treated groups. In the meantime, the mice in the experimental groups were orally gavaged JT3-1, FBL-3a, or ZLP-4b at 1 × 10^8^ colony forming units per day for a continuous 2 weeks, respectively, while the control mice were given the same volume of phosphate-buffered saline. During the experiment, abnormal performance in mice was recorded, such as death, diarrhea, loss of appetite, weight loss, and so on.

### Production Performance Analysis

In this test, the body weight of all mice were recorded at the same time every day and under identical conditions. Main organs such as the heart, lung, liver, kidney, thymus, and spleen were weighed by an electronic balance after euthanasia. The weight/body weight of an organ was expressed as organ index.

### Histological Staining

The duodenum, jejunum, ileum, and colon tissues were dipped in 4% paraformaldehyde solution (Servicebio Co., Ltd., Wuhan, China) and stained using hematoxylin and eosin (H&E) solution. Images of morphological character were captured at ×40 and ×100 magnification by using a scanning electron microscope SU8100 (Hitachi Co., Ltd., Japan). The intestinal villus height, crypt depth, and the rate of villus height to crypt depth were calculated by CaseViewer 2.0 software.

### Cytokine Profiling Assay

Essential cytokines in the serum of mice such as IL-4, IL-6, IL-10, IL-17A, and TNF-α were determined with Mouse ProcartaPlex Panel (Thermo Fisher Scientific, Waltham, USA) following the instructions from the manufacturer. The samples were read by Luminex TM 100/200TM instrument (Luminex Corp, Austin, TX, USA).

### Fecal Extraction and 16S Sequence Analysis

Fresh feces in colon were collected under sterile conditions. The feces were promptly frozen in liquid nitrogen and then preserved at −80°C for the next step. The bacterial genomic DNA was extracted from the stored fecal pellets using QIAamp Fast DNA Stool Mini Kit (Qiagen, Germantown, MD) according to the manufacturer. The V3–V4 region of the bacterial 16S rRNA gene was PCR-amplified using the forward primer 5′-CCTACGGGNGGCWGCAG-3′ and reverse primer 5′- GACTACHVGGGTATCTAATCC-3′. The PCR product purification and amplicon library construction were performed by the Illumina NovaSeq PE250 platform at a commercial company (LC-Bio Technology Co., Ltd, Hang Zhou, China) according to the standard protocols.

### Statistical Analysis

Figures were generated using GraphPad Prism 6.0 software. The statistical significance in our study was determined by *t*-test and χ^2^ test by the SPSS statistical program, and *P* < 0.05 was considered statistically significant. The analysis was repeated for three times independently.

## Results

### Production Performance and Organ Index Analysis

**Figure 1:**
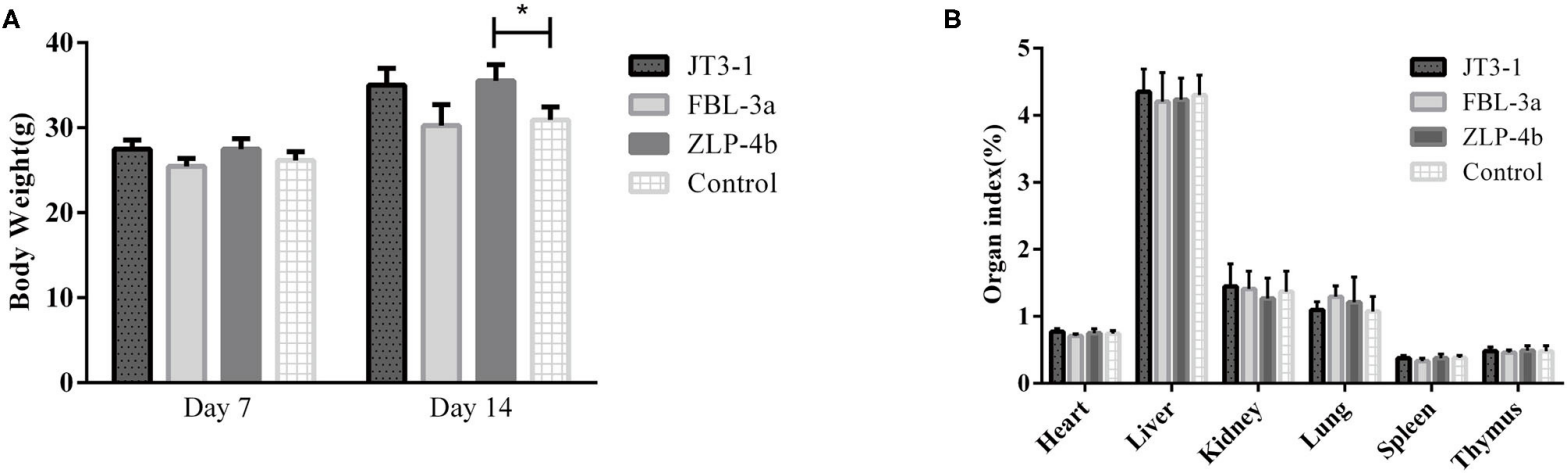
Probiotic supplementation improved the overall performance of mice. **(A)** The body weight of mice at days 7 and 14, respectively. **(B)** Organ indexes analysis of the heart, liver, and spleen of mice in all the groups. The data are presented as mean ± SD. The values indicate mean ± SD. *P < 0.05.

During the experiment period, no aberrant behavior was found. The overall performance in all three groups of the mice that received probiotics was improved compared to the control group. As shown in Figure 1A, there was no difference in the body weight on days 7 and 14 in the experimental and control groups, except that the weight of the mice treated with ZLP-4b was significantly increased than that of the control group on day 14 (Figure 1A). No significant difference was observed in the weight of the heart, liver, spleen, lung, thymus, and kidney between the three probiotic-administrated groups and the control group (Figure 1B). The results indicated that probiotic JT3-1, FBL-3a, and ZLP-4b administration did not affect the growth performance of mice.

### Probiotic Supplementations Improve the Intestinal Mucosa Morphology

**Figure 2:**
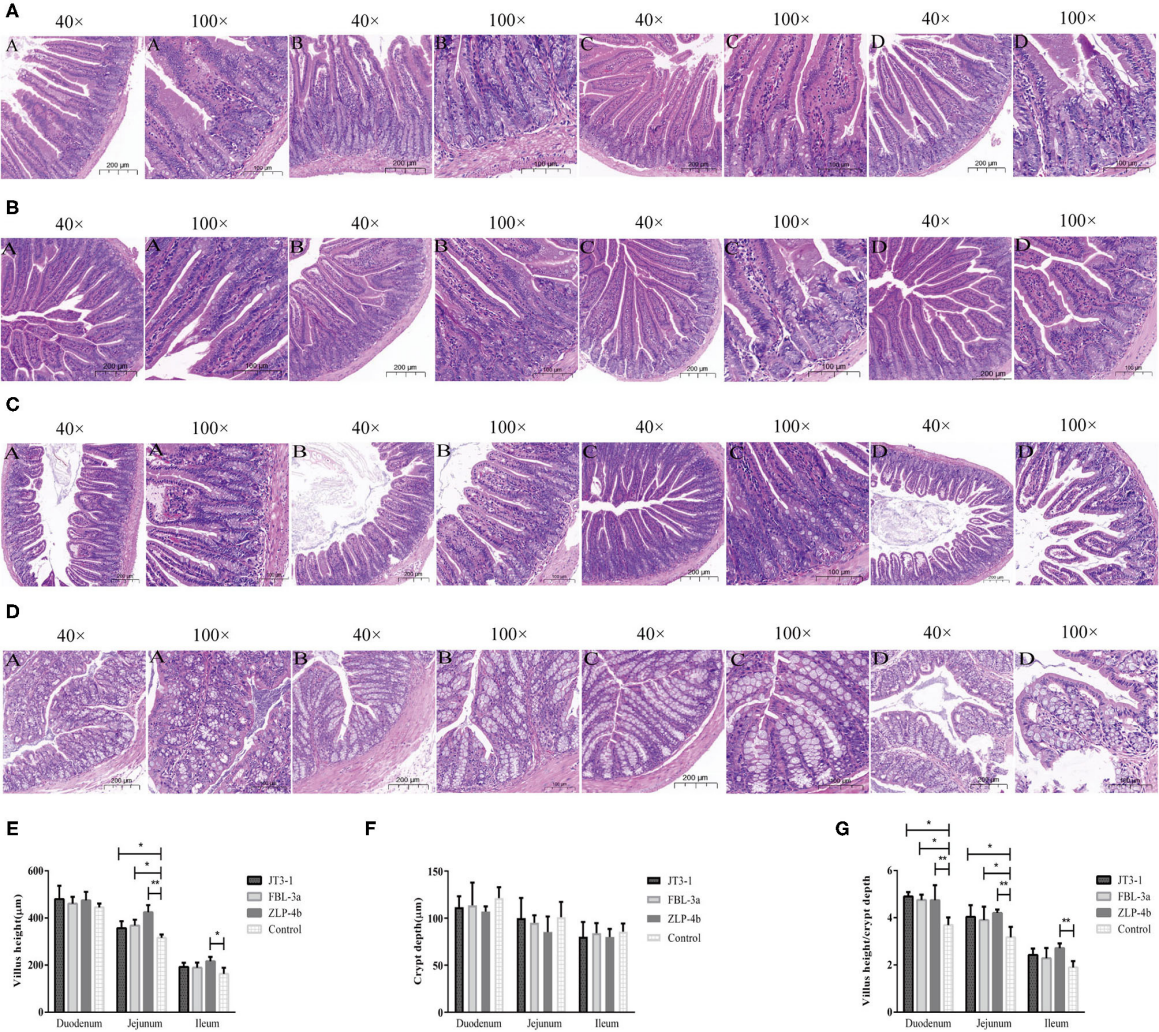
The effects of probiotic pretreatment on the epithelial mucosa integrity of the intestine of mice. **(A–D)** Intestinal morphology shown by hematoxylin and eosin staining of the duodenum, jejunum, ileum, and colon tissues of mice. The images of intestinal morphology at ×40 and ×100 magnification are shown, respectively. **(E)** Statistical analysis of villus height (μm), **(F)** crypt depth (μm), and **(G)** the ratios of villus height (μm) to crypt depth (μm) in the duodenum, jejunum, and ileum of mice, respectively. **(A)** JT3-1 group, **(B)** FBL-3a group, **(C)** ZLP-4b group, and **(D)** control group. *P < 0.05; **P < 0.01.

This study was aimed to investigate whether these isolated strains contribute to the intestinal mucosa function of mice. It was found that the three probiotic administration groups significantly improved the intestinal epithelial mucosal integrity in the jejunum, ileum, and colon tissues but not the duodenum compared to the control mice (Figures 2A–D). Meanwhile, the villus heights and the ratios of villi heights to crypt depths in these tissues were also increased (Figures 2E–G). These results showed that the three probiotic-administrated groups presented an improved intestinal mucosal integrity.

### Effects of Probiotics on Cytokine Modulation

**Figure 3:**
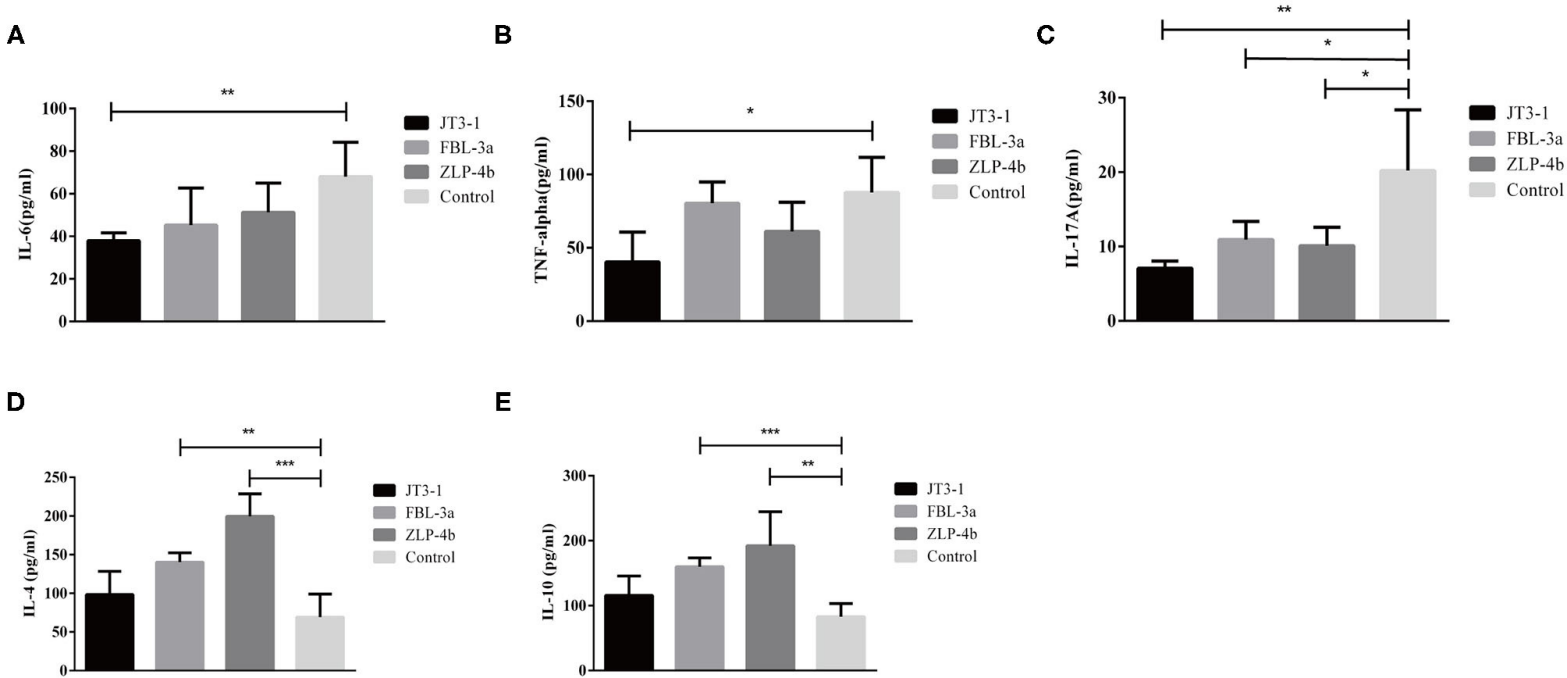
Effects of the oral administration of probiotics on cytokine modulation. **(A–E)** IL-6, TNF-α, IL-17A, IL-4, and IL-10 level in the serum of mice. Data are expressed as mean ± SD. *P < 0.05; **P < 0.01; ***P < 0.001.

The serum anti-inflammatory cytokines (IL-4 and IL-10) and pro-inflammatory cytokines (IL-6, IL-17A, and TNF-α) were measured to indicate the inflammatory levels in mice after probiotics were used. The IL-10 and IL-4 levels were dramatically enhanced in the FBL-3a and ZLP-4b groups, except in the JT3-1-treated group (*P* < 0.01 or *P* < 0.001, Figures 3D,E). The content of IL-6 and TNF-α showed a reduction in the JT3-1-treated group compared to the control mice; a similar decrease was also found in FBL-3a and ZLP-4b-mice, although the changes were not distinct (*P* < 0.01, *P* < 0.05, or *P* > 0.05, Figures 3A,B). In addition, Figure 3C revealed that the content of IL-17A in sera was reduced after the mice received probiotics compared to the control group (*P* < 0.05, Figure 3C). It seemed that *B. velezensis* JT3-1 preferred to inhibit pro-inflammatory cytokines, while *L. plantarum* FBL-3a and *L. salivarius* ZLP-4b were more inclined to stimulate the production of anti-inflammatory cytokines.

### Characterization of the Gut Microbiota in Mice After Probiotic Administration

**Figure 4:**
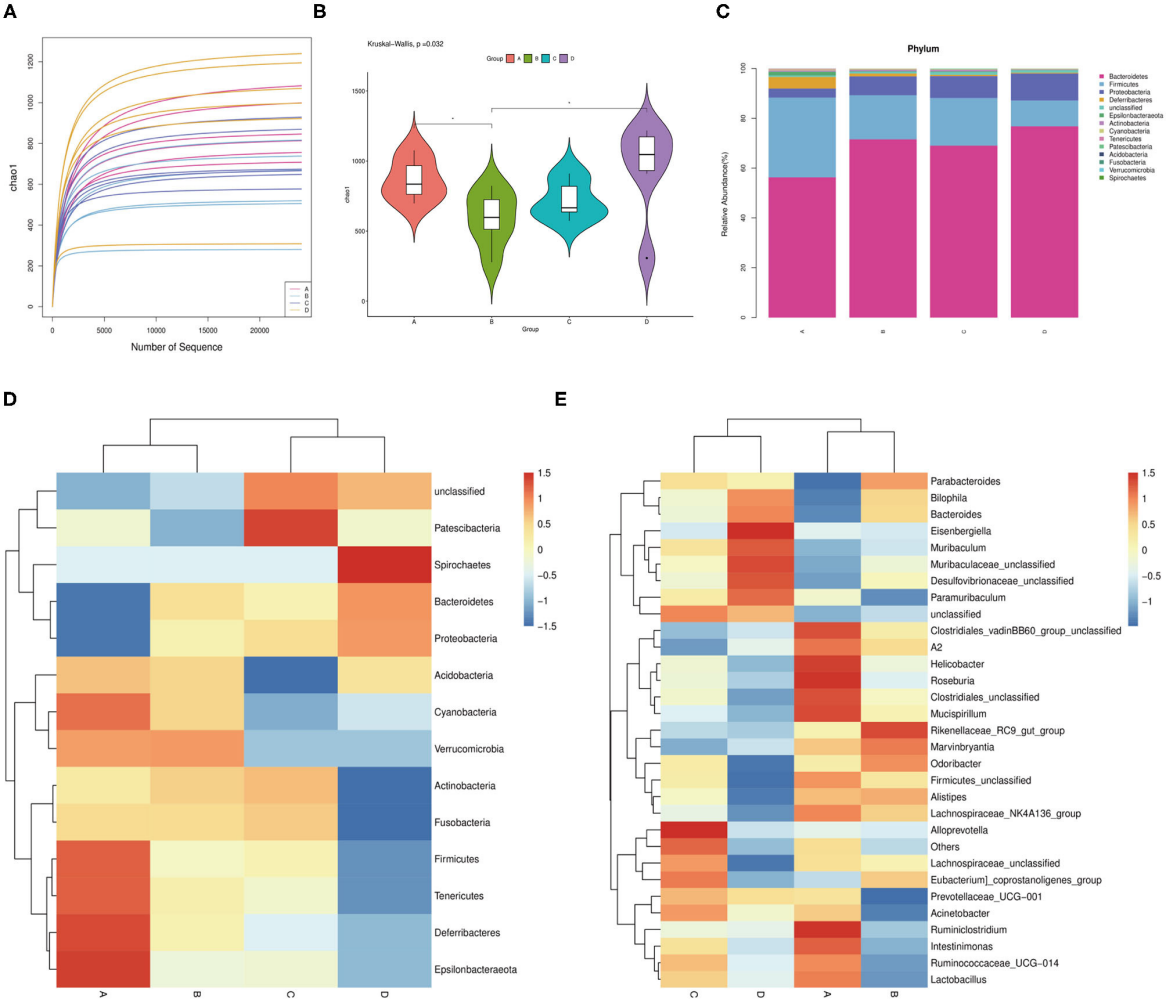
Microbial community changes in the colon content of mice after probiotic administration. **(A)** Bacterial rarefaction curves for assessing the sequencing depth of each sample. **(B)** Alpha diversities in bacterial communities as determined by Chao1 index. **(C)** Average relative abundances of taxa at the phylum level. **(D)** Heat map and hierarchical clustering of phylum in the intestinal bacterial communities of mice. **(E)** Clustering heat map analysis of bacterial genera at the genus level. **(A)** JT3-1 group, **(B)** FBL-3a group, **(C)** ZLP-4b group, and **(D)** control group.

The effects of probiotic administration on the intestinal microbial communities in mice were estimated by 16S rDNA sequence. The rarefaction curves showed that nearly all the bacteria species were sequenced in the feces of mice (Figure 4A). It was found that the mice possessed a lower bacterial species richness compared with the control group after the oral administration of probiotics (Figure 4B). Moreover, the detection of bacterial phyla displayed that the relative abundance of Firmicutes was significantly increased, while the relative abundance of Proteobacteria and Bacteroidetes markedly declined in the fecal microbiota from the probiotic-treated mice (Figure 4C). Besides this, some phyla such as Deferribacteres, Epsilonbacteraeota, Fusobacteria, and Actinbacteria showed a higher abundance, while lower Spirochaetes was observed in the probiotic-treated group (Figure 4D). At the genus level, the bacterial genera in the fecal microbiota showed that the relative abundance of *Bilophila* and *Bacteroides* was reduced, while that of *Alistipes* was increased in the probiotic supplementation group compared with that in the control group. Strangely, the *Lactobacillus* abundance in JT3-1 and ZLP-4b group was raised, but the FBL-3a-treated group was reduced (Figure 4E).

## Discussion

Antibiotics had a profound impact on bacterial infection in the therapeutic treatment of diseases (18). However, a number of serious problems also appeared subsequently as the synthesis of antibiotics in large quantity, such as the threat of bacterial resistance, antibiotic-associated diarrhea, and super-infection. In this study, we identified that three candidate probiotic strains from Northwest China improved the immune function and gastrointestinal health of mice. Firstly, no exceptional changes of body weight and organ index both in the control group and the treated groups demonstrated the safety of these three probiotics during the oral administration period. Secondly, the length of the villi and the villus length/crypt depth ratio were improved, and the integrity of the intestinal epithelial mucosa in the ileum and colon tissues was ameliorated, except the duodenum and jejunum segments, compared to the control mice after probiotic gavage. A similar result reported that probiotic supplementation enhanced the height of villus and the depth of crypt (19). Maybe it is just because microorganisms mainly colonize the back part of the intestine, particularly in the colon where microbes act as a major modulator of the immune system of the mucosa (20).

Cytokines play a considerable part in the immune modulatory and defense system of the host (21). However, not all probiotics could suppress pro-inflammatory cytokines and enhance anti-inflammatory factors—for instance, *Escherichia coli* strain Nissle 1917 had both pro- and anti-inflammatory effects because of the fact that *E. coli* Nissle 1917 also benefits from the inflamed environment, and it could account for why its anti-inflammatory effects are mild to moderate (22, 23). IL-4 and IL-10 play an important role in the immune modulatory pathway of the host as acknowledged inflammatory suppressor, and IL-10 production depends on IL-4 (24, 25). The cooperation between IL-10 and IL-4 suppressed Th1-related parameters (26). IL-4 is required for the induction of the class switch to IgG1 antibodies in cardiolipin-specific B cells (27). A previous study reported that the level of IL-4 was significantly evaluated after supplementation of a probiotic mixture of *L. paracasei* and *L. fermentum* (28). Some research data confirmed that IL-10 had a protective role in epithelial cells (29). In addition, IL-10 plays an effective role in inhibiting inflammation and pathogen clearance during *Borrelia recurrentis* infection (30), and *L. lactis* producing IL-10 could be a therapy of murine colitis (31). Similar effects were also found in our research that probiotic administration significantly enhanced IL-4 and IL-10 content in the FBL-3a and ZLP-4b groups. It suggested that FBL-3a and ZLP-4b have anti-inflammatory potential to some extent.

IL-6, TNF-α, and IL-17A are important pro-inflammatory mediators that play a crucial role in the processes of inflammation (32). IL-6 and TNF-α gene expression was upregulated in most intestinal inflammations such as IBD, colitis, and necrotizing enterocolitis (33–35). In addition, IL-6 exerts an anti-inflammatory role in a pancreatitis model by regulating the generation of cytokines, expression of adhesion agents, and activation of neutrophils (36). Two *B. subtilis* strains (BS1 and BS2) could decrease the content of TNF-α and IL-6 in a mice model (13). IL-23 promoted the pro-inflammatory cytokines IL-6 and IL-17 in autoimmune inflammatory disease models (37). IL-17A played a pivotal part in the development of dextran sodium sulfate-induced colitis by regulating the balance of Th17 and Treg cells (38). A recent research revealed that *L. helveticus* and *L. rhamnosus* suppressed interleukin 17 transcription in *Citrobacter rodentium*-induced colitis in mice (39). In this study, we found that *B. velezensis* JT3-1 could diminish the content of TNF-α, IL-6, and IL-17A, while *L. plantarum* FBL-3a and *L. salivarius* ZLP-4b merely restrained the IL-17A level. Such results are in accordance with the previous studies, implying that probiotics obtained from livestock in Northwest China exerted a protective and salutary function in mice *via* modulating cytokine secretion.

Gut microbes play a substantial role in the maintenance of intestinal barrier function and modulation of the immune pathway (21), and the diversity of the gut microbiota is significant to the health of the hosts. We found that the species of bacteria in the gut decreased to a certain extent after probiotic administration, but some positive changes also emerged. The increase of Firmicutes/Bacteroidetes ratio contributes to nutrient intake and energy transformation (40). Proteobacteria include a lot of pathogenic germs such as *Salmonella, E. coli, Vibrio cholera*, and *Helicobacter* bacteria which can cause many infectious diseases (41). In Spirochaetes, many pathogens were identified as causes of many illnesses—for instance, *Leptospira* (leptospirosis), *Borrelia burgdorferi* (Lyme disease), *Treponema pallidum* (syphilis), *Treponema carateum* (pinta), *Borrelia recurrentis* (relapsing fever), and *Treponema pertenue* (yaws) (42–44). In this study, we found that the relative abundance of these two bacteria (Proteobacteria and Spirochaetes) was reduced by isolated probiotic pretreatment. *Bilophila* can activate Th1 cells to promote the production of IFN-γ, but it causes appendicitis as opportunistic pathogens (45). In our study, the abundance of *Bilophila* is reduced after probiotic supplementation. *Lactobacillus* is negatively correlated with TNF-α; maybe it is why the FBL-3a group with few *Lactobacillus* appeared to have higher levels of TNF-α than the other groups (46).

In our laboratory, before the animal experiment, many *in vitro* tests were conducted to assess the antibacterial activity and safety of JT3-1, FBL-3a, and ZLP-4b, including bile salt acid and tolerance tests, antibacterial tests, hemolytic activity tests, and antibiotic susceptibility assay (47–49). Our results demonstrated that *Bacillus* and *Lactobacillu*s isolated from livestock in Northwest China can confer benefits to mice by improving the intestinal mucosa integrity and modulating the cytokines linked to inflammation and immunity. Moreover, probiotic administration exerted positive effects on the gut microbiome of mice. This work supported the potential for *B. velezensis* JT3-1 and *L. salivarius* ZLP-4b to be functional probiotics based on their capacity of beneficial modulation of the intestinal morphology and immune-related cytokines. Further research should be performed before these strains are served in clinical practice.

## Data Availability Statement

The datasets presented in this study can be found in online repositories. The names of the repository/repositories and accession number(s) can be found below: https://www.ncbi.nlm.nih.gov/genbank/, CP032506; https://www.ncbi.nlm.nih.gov/genbank/, CP034694; https://www.ncbi.nlm.nih.gov/genbank/, CP062071.

## Ethics Statement

The animal study was reviewed and approved by Lanzhou Veterinary Research Institute, Chinese Academy of Agricultural Sciences. Written informed consent was obtained from the owners for the participation of their animals in this study.

## Author Contributions

YiL and YoL designed this study and critically revised the manuscript. DJ, JiaW, HL, and XY performed the experiments and data analysis. JLi, JinW, GG, JLu, SX, and HY participated in the coordination and manuscript revision. All authors read and approved the final manuscript.

## Funding

The authors would like to acknowledge the National Key Research and Development Program of China (2018YFD0501804, 2018YFD0502305, and 2017YFD0501200), the Agriculture Research System of MOF and MARA (CARS-37), and the Jiangsu Co-innovation Center program for the Prevention and Control of Important Animal Infectious Diseases and Zoonosis for the funding support.

## Conflict of Interest

The authors declare that the research was conducted in the absence of any commercial or financial relationships that could be construed as a potential conflict of interest.

## Publisher's Note

All claims expressed in this article are solely those of the authors and do not necessarily represent those of their affiliated organizations, or those of the publisher, the editors and the reviewers. Any product that may be evaluated in this article, or claim that may be made by its manufacturer, is not guaranteed or endorsed by the publisher.
